# Genome Editing for Sustainable Crop Improvement and Mitigation of Biotic and Abiotic Stresses

**DOI:** 10.3390/plants11192625

**Published:** 2022-10-06

**Authors:** Mohd Fadhli Hamdan, Chou Khai Soong Karlson, Ee Yang Teoh, Su-Ee Lau, Boon Chin Tan

**Affiliations:** 1 Centre for Research in Biotechnology for Agriculture, Universiti Malaya, Kuala Lumpur 50603, Malaysia; 2 Department of Cell and Molecular Biology, Faculty of Biotechnology and Biomolecular Sciences, Universiti Putra Malaysia, Serdang 43400, Malaysia

**Keywords:** biotechnology, climate change, CRISPR, crop improvement, genome editing

## Abstract

Climate change poses a serious threat to global agricultural activity and food production. Plant genome editing technologies have been widely used to develop crop varieties with superior qualities or can tolerate adverse environmental conditions. Unlike conventional breeding techniques (e.g., selective breeding and mutation breeding), modern genome editing tools offer more targeted and specific alterations of the plant genome and could significantly speed up the progress of developing crops with desired traits, such as higher yield and/or stronger resilience to the changing environment. In this review, we discuss the current development and future applications of genome editing technologies in mitigating the impacts of biotic and abiotic stresses on agriculture. We focus specifically on the CRISPR/Cas system, which has been the center of attention in the last few years as a revolutionary genome-editing tool in various species. We also conducted a bibliographic analysis on CRISPR-related papers published from 2012 to 2021 (10 years) to identify trends and potential in the CRISPR/Cas-related plant research. In addition, this review article outlines the current shortcomings and challenges of employing genome editing technologies in agriculture with notes on future prospective. We believe combining conventional and more innovative technologies in agriculture would be the key to optimizing crop improvement beyond the limitations of traditional agricultural practices.

## 1. Introduction

Climate change, such as extreme weather or temperature, drought, increasing soil salinity, and flooding, significantly affects the food production system, posing serious threats to food security. The adverse effects of climate change on agricultural productivity have been reported in several regions, including Asia [1], sub-Saharan Africa [2], and the European Union (EU) [3]. For example, the heatwave and drought in the EU in 2018 have reduced cereal production by 8% compared to the previous five-year average [4], causing fodder shortages for livestock and increasing commodity prices. The impacts of climate change on agriculture in developing countries are more significant than in developed countries, mainly as these countries are located in tropical latitudes, which are more sensitive to climate change [5]. In addition, differences in vulnerability between these regions might be due to differences in endowments of human skills, physical infrastructure, and rapid demography growth, causing developing countries to have lower levels of resilience [6,7,8]. Ensuring sustainable crop production and food security has become challenging not only due to the growing environmental pressures but also the ever-increasing human population. Around 720 to 811 million people, about a tenth of the global population, still suffer from hunger. Meanwhile, more than 2 billion people are in the ‘food insecure’ category [9]. Another 130 million people may be added to the latter category due to the recent COVID-19 pandemic [10]. These problems will continue to worsen with the projected global population growth since the yield of grain crops, such as rice, wheat, and maize, has already reached a plateau [11]. With an estimated world population of 9.7 billion by 2050, crop productivity will need to increase by another ~70% while simultaneously reducing the environmental impacts [12]. Moreover, climate change increases the severity of biotic and biotic stresses on crops. Biotic stresses, such as pathogens, insect pests, and weeds, cause average output losses ranging from 17.2% in potatoes to 30.0% in rice [13]. Likewise, abiotic stresses, such as temperature extremes, drought, and lack of nutrient deficiency, caused the loss of 51–82% of the global crop output annually [14]. As the intensity of biotic and abiotic stresses on crops increases because of climate change, novel approaches are required to enhance plant tolerance. Given that the conventional agricultural practices are inadequate to meet current and future food demands and deal with the aggravated impacts of biotic and abiotic stresses due to climate change, developing practical and effective adaptation strategies is indispensable to enhance crop productivity and ensure food security. Ideally, the strategies driving this effort should be sustainable and environmentally friendly while minimizing adverse environmental impacts.

Crop breeding, including cross-breeding and mutation breeding, has been used to enhance crop performance under climate change scenarios. However, breeding programs can be laborious and time-consuming, even aided by marker-assisted selection. It can take 8 to 10 years [15] or 6 to 15 years [16] to produce a genetically superior cultivar for agricultural production. Plant breeders have used cross-breeding based on naturally occurring mutations [15] or mutation breeding techniques based on ionizing radiation and chemical mutagens to generate new varieties with desired agronomic traits, including improved stress-tolerance potential and biofortification [17]. Nevertheless, since cross-breeding is limited to traits present in the parental genomes, low variability in elite germplasms restrains the use of this technique. The outcomes of the mutation breeding technique are unpredictable even though lower mutation rates have been reported in essential genes compared to non-essential genes [18]. In addition, complex and tedious screening and selection procedures are required to identify the desired trait from a large population of mutagenized plants [19]. Transgenic technologies that involve transferring desired trait-coding genes into the elite cultivars are undoubtedly an alternative to counter losses in crop yield [20]. However, the time and expenses for developing a genetically modified (GM) crop with desirable traits are enormous. The major limitation of this method is the low public acceptance of GM crops and, related to this, the complex and strict safety regulatory procedures [21]. In addition, different countries have adopted different regulatory procedures. However, to date, only a few countries, such as Switzerland, strictly restricted or legally prohibited the cultivation of GMOs [22].

Given the importance of securing sustainable crop yield, the challenge now is to improve the existing technologies or develop alternative technologies/solutions to increase crop yields. Here, we discuss the possibility of using genome editing, particularly the CRISPR/Cas9 system, to alleviate the impact of environmental stress and enhance crop production. A bibliometric analysis of CRISPR-related articles published in the SCOPUS database was done to evaluate its current trend of publications from 2012 to 2021. The selected timeline represents the first decade since the discovery of CRISPR/Cas9 in 2012 for use in genome editing [23]. This content analysis allows us to identify certain ‘hot spots’ or themes and reveal the potential of CRISPR-related research in plants.

## 2. Genome Editing Technologies

**Figure 1:**
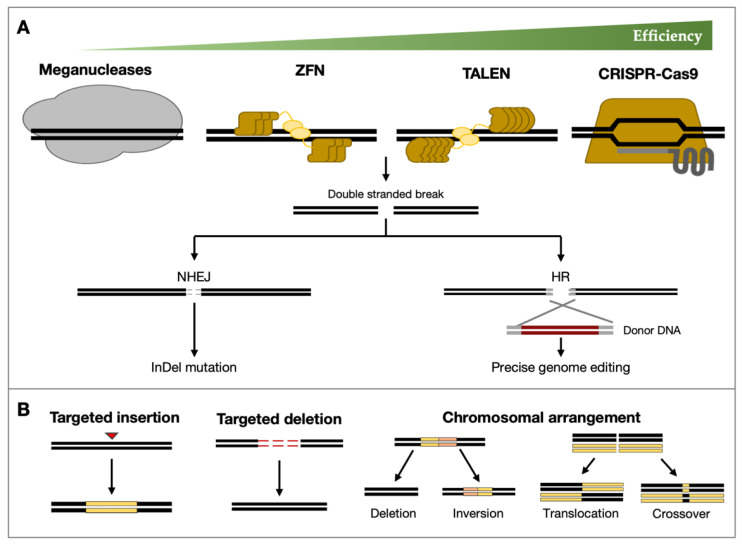
Different types of sequence-specific nucleases and types of editing. (**A**) Meganucleases, zinc finger nuclease (ZFN), transcription activator-like effector nuclease (TALEN), and CRISPR/Cas9 induces double-stranded breaks, which were corrected by non-homologous end-joining (NHEJ) and homologous recombination (HR). (**B**) Schematic diagram of target insertion, target deletion, and chromosomal arrangement through genome editing technologies. InDel, insertion-deletion.

Genome editing techniques using sequence-specific nucleases (SSNs) have become popular in plant research. They have been used to develop high-yielding crops, improve the adaptability of crops to environmental stresses or enhance their nutrition content [24]. To date, there are four SSNs, namely meganucleases, zinc finger nucleases (ZFNs), transcription activator-like effector nucleases (TALENs), and clustered regularly interspaced palindromic repeat (CRISPR)/CRISPR-associated (Cas) protein systems (Figure 1). These technologies allow precise targeting and modifying of specific DNA sequences in three common steps: (1) an exogenous engineered nuclease consisting of a recognition module and nuclease domain recognizes the target DNA sequence, (2) the engineered nuclease binds to the target DNA sequence and induces double-strand breaks (DSBs) at or in the vicinity of the target site and (3) the DSBs will then be repaired by either non-homologous end-joining (NHEJ) or homologous recombination (HR). NHEJ is an error-prone repair mechanism that often results in insertion and deletion (Indel) mutations, whereas HR results in a precise repair of DSBs [25].

Meganucleases were the first SSN used to create targeted DSBs in eukaryote genomes [26]. They are naturally occurring endonucleases found in prokaryotes, archaea, and unicellular eukaryotes [27]. The first meganuclease, *I*-*SecI*, was discovered in yeast [28]. Meganucleases are the most specific naturally occurring endonucleases as they recognize 14–40 bp long DNA sequences [29]. These enzymes have a larger recognition site than the type II restriction enzymes used in recombinant DNA technology. Due to their long recognition sequence and high specificity, meganucleases can efficiently target and modify any sequence of interest [30]. For these reasons, meganucleases have been used to create targeted DSBs in eukaryote genomes since the 1990s. In 1993, Puchta and colleagues published a landmark paper demonstrating that *I*-*SecI*-induced DSBs enhance HR in *Nicotiana tabacum* [31]. This marked the arrival of precise genetic engineering in plants using SSNs. Since then, several efforts have been made to introduce trait genes into plants. For example, D’Halluin et al. [32] inserted multiple trait genes, 4-hydroxyphenylpyruvate dioxygenase (*hppd*) and modified enol-pyruvylshikimate-3-phosphate synthase genes (*epsps*) into cotton using meganucleases to enhance its herbicide tolerance. Although meganucleases have been successfully applied for targeted gene editing in the plant, they have a few limitations, such as the low catalytic activity of the enzyme, prone to sequence degeneracy, and the lack of mature DNA binding structure, which hinders their wide applications [33,34].

ZFNs are fusions of the DNA recognition domain of zinc finger protein and the cleavage domain of the FokI endonuclease [35]. ZFNs act through DNA/protein recognition, and each zinc finger recognizes three base pairs (bp). As FokI must dimerize to become active, ZFNs should be designed as a pair to ensure the correct orientation and appropriate spacing for FokI dimerization [36]. To date, ZFN-mediated gene modification has been reported in various crops, such as soybean [37], maize [38], wheat [39], and rice [40]. Yet, their application as an editing tool in crops is limited because of the complexity and cost of the protein construction for each targeted site, and the potential cytotoxicity effects, presumably due to cleavage at off-target sites [41].

Similar to ZFNs, TALENs comprise transcriptional activator-like effector (TALE) repeats (comprise the DNA binding domain) and a FokI endonuclease (comprises the cleavage domain) [30]. TALEs are type III effector proteins derived from *Xanthomonas* spp. Their DNA binding ability was first reported in plants in 2007 [42,43]. In 2009, the recognition code of TALE targeting DNA sequence was also decrypted [44]. The DNA binding domain in TALE monomers contains a central repeat domain, which consists of tandem repeats of 34 amino acid residues. Each 34-amino-acid-long repeat in the central repeat domain targets only one nucleotide in the target DNA sequence. This made TALENs a better gene-editing tool compared to ZFNs as they allow flexible target design. Two hypervariable amino acid residues at the 12th and 13th positions are highly variable (termed as repeat variable di-residue [RVD]) and critical for specific nucleotide recognition.

TALENs have been demonstrated in various plant species, such as Arabidopsis, tobacco, soybean, sugarcane, maize, and wheat [45,46]. The use of TALENs in crop improvement was first reported in rice, where *OsSWEET14* (bacterial blight susceptibility gene) was disrupted, and the resulting mutant rice displayed bacterial blight resistance [47]. Other applications of TALENs in crop improvement include producing flavor in rice [48], developing powdery mildew resistant wheat [49], enhancing the nutrient content of soybean [50], and increasing anthocyanin levels in tomatoes [51]. However, despite their potential for crop improvement, several challenges of TALENs have limited their applications. One major drawback is the inefficient delivery of the TALEN system into a cell due to the large size of cDNA encoding TALEN (about 3 kb). Furthermore, the construction of TALE repeats remains a bottleneck and the efficiency of TALENs targeting a gene is variable [45].

The discovery of the CRISPR/Cas9 genome editing system has revolutionized the fields of functional genomics in animal and plant biology. Originating from bacteria and archaea as an adaptive immunity system, the CRISPR/Cas9 system has become a viable tool for targeted genome editing in prokaryotes and eukaryotes.

## 3. The CRISPR/Cas System

In 1987, CRISPR was discovered accidentally in the *Escherichia coli* genome while Ishino et al. [52] were sequencing the *iap* gene encoding alkaline phosphatase isozyme conversion enzyme. Downstream the *iap* gene, the authors discovered a unique set of tandemly repeating 29-nucleotide (nt) DNA sequences interspersed with 32-nt spacer sequences. They were unaware of the biological role of these repeats due to the lack of sequence homology with other known sequences. Later in 1993, long tandem repeats were discovered by Mojica et al. while sequencing several *Haloferax mediterranei* genome segments [53]. This marks the first finding of archaeal direct repeats. The series of interspaced repetitions was later classified as clustered regularly interspaced short palindromic repeats (CRISPR) [54]. As biological science advances towards the genomic era, CRISPR has now been identified in various bacterial and archaeal genomes.

In 2005, it was finally revealed that spacers present within CRISPRs were produced by invading phages and plasmids [55,56]. This established the CRISPR/Cas system as an adaptive immune system of bacteria and archaea that defends bacteria from bacteriophages and mobile genetic elements (MGEs) by eliminating invasive genomic elements [57]. CRISPRs can prompt the capture of invading DNA fragments to serve as a record of prior genetic aggressions [55,56]. The significance of CRISPR/Cas systems as adaptive immunity has been reinforced by subsequent experimental findings, which reiterated that new spacer sequences from the infecting phage are acquired by the bacterial CRISPR array [58,59,60,61].

The CRISPR/Cas systems were initially classified into three types (Types I, II and III) based on proteins and accessory RNAs. Type I and III systems use a complex of multi-Cas protein for target DNA recognition and cleavage, whereas the Type II system relies on a single Cas9 protein to accomplish the interference [62]. Further experimentation and analysis have further divided the classification into 2 classes, 6 types and 33 subtypes [63]. As the classification of CRISPR has been described in earlier reviews [64,65], we are not explaining it in detail in this paper.

The type II CRISPR/Cas system is the most widely used and best studied due to its straightforward constructs compared to the other systems. Type II CRISPR/Cas system employs a single protein, Cas9, and two non-coding RNAs, CRISPR RNA (crRNA) and trans-activating crRNA (tracrRNA), for target recognition and cleavage. The dualtracrRNA:crRNA guides the Cas9 nuclease to recognize protospacer adjacent motifs (PAMs or 5′-NGG-3′) on the target DNA sequence. Cleavage of the target DNA is then performed by two Cas9 nuclease domains, the HNH domain (cleave the DNA strands complementary) and RuvC-like domain (cleave the non-complementary). Both induce a DSB three bases upstream of the PAM site of the target region.

The newly established CRISPR/Cas system replaces the dual-tracrRNA:crRNA with a single guide RNA (sgRNA) to ease the genome modifications. With this system, one can perform genome editing by simply modifying the 20-nucleotide sgRNA to be complementary to the target DNA. Overall, a CRISPR/Cas9 project involves the steps below:

(i) Target and PAM sequence identification;

(ii) Evaluate off-target effects;

(iii) sgRNA synthesis;

(iv) Cloning of the sgRNA into a suitable plant expression plasmid;

(v) Plant transformation and screening of the edited lines.

As described above, CRISPR-mediated genome editing involves the generation of Cas9-induced DSBs that are repaired by NHEJ or HR. For developing agronomic traits, precise genome modification is required. Although HR can be used to precisely repair the DSBs when DNA donor templates are supplied, it is rarely used in crop improvement because of its low efficiency in higher plants. The recent developed powerful technologies, i.e., base editing and prime editing, have partly overcome such barriers and greatly improved crop breeding opportunities.

Base editing (BE) is a technique that directly converts one target genomic DNA base into another at a targeted locus without producing a double-stranded break. Combining cytosine or adenine deaminases with CRISPR-Cas9, a range of cytosine base editors (CBEs) and adenine base editors (ABEs) has been developed in recent years [66,67,68]. These varieties allow exact C-to-T or A-to-G base conversions without causing a DSB. Using CBE, cytosine (C) is deaminated to create uracil (U). The uracil (U) is read as thymine during DNA replication (T). CBE consequently provides a single-base substitution from CG to TA [69]. In ABE, the inactive CRISPR–Cas9 domain is connected to adenosine deaminase, which helps convert adenine (A) to inosine, unlike cytidine deaminase in CBE. This inosine is read during DNA replication as guanine (G). Consequently, ABE generates AT to GC base substitutions [70]. Since their discovery, base editors have become valuable tools for precisely modifying the genomes of eukaryotic organisms [71,72,73,74].

Prime editing (PE) is another innovation made to the genome editing toolbox. Previous BE procedures created single base substitutions for four transitions (C > T; T > C; A > G; G > A), and newer studies included two transversions (C > G and G > C). Instead of a deaminase, PE uses an extended guide RNA (pegRNA)-guided reverse transcriptase, which allows pegRNA to install substitutions, insertions, and deletions [75]. In contrast, PE contains all 12 alterations, including the eight transversions. This increases the versatility and robustness of the gene editing strategy. Although still in its infancy, PE applications show promise in multiple cell types, organoids, and mice embryos. Data on its application in plant systems have also started to emerge. In maize, PE has introduced W542L and S621I double mutations in two ALS genes, *ZmALS1* and *ZmALS2*, which may confer resistance to several ALS-inhibiting herbicides. In rice, the level of PE efficiencies ranged from 2.22 to 31.3% [76]. In one experiment, triple amino acid substitutions (T169I, A170V, and P173S) were introduced into *OsEPSPS* [77], which may confer a higher level of glyphosate resistance [78].

In addition to the Cas9 protein, three more family proteins, namely Cas12, Cas13, and Cas14, as well as their orthologs, have been identified. Cas12 family protein is considered more advanced and versatile than Cas9 due to several characteristics, such as smaller size, lack of need for trans-activating crRNA (tracrRNA), and ability to cleave DNA via its RuvC domain. In addition, it can edit many genes from a single RNA transcript due to an intrinsic RNAse that can process its own guide RNA array [79,80,81]. Type IV Cas13 has an RNA-guided RNase domain that could degrade nearby single-stranded-RNA (ssRNA) molecules [82]. It has been applied for targeted RNA interference in various organisms, including animals, human viruses, and plants [83]. Cas14 is exceptional for sequence detection since it does not need a PAM and is particularly sensitive to mismatches in the center of its target region [84].

**Figure 2:**
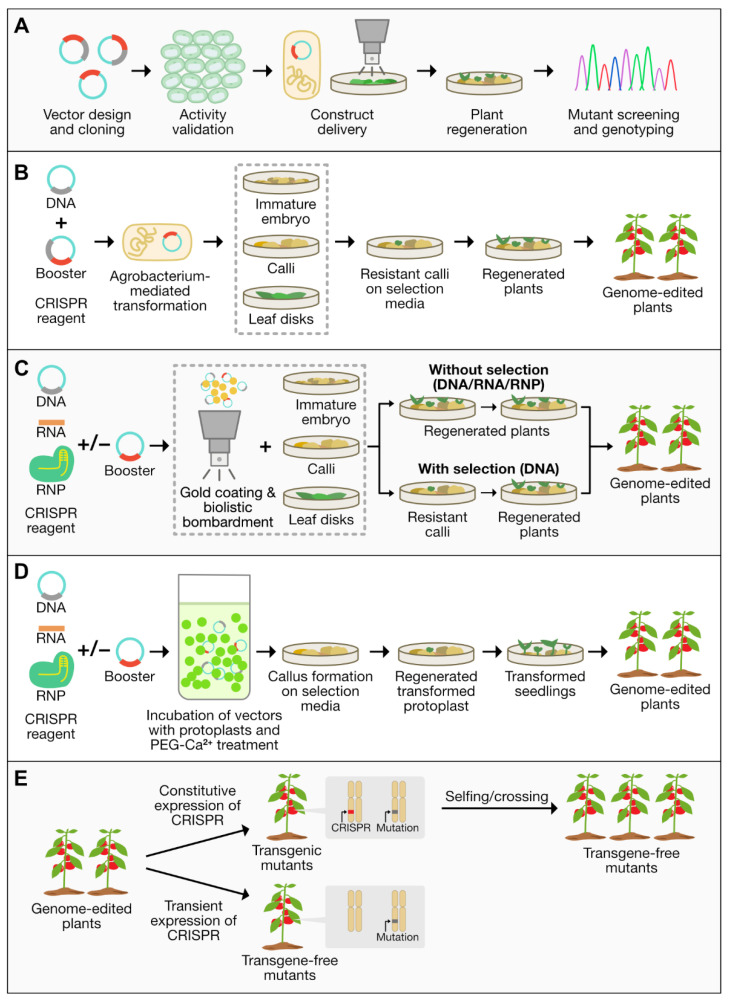
General procedures in plant transformation, delivery methods of CRISPR cargo, and transgene-free mutant development. (**A**) Major steps in plant genome editing. Once transformation vectors are designed and constructed, their activity may be validated with protoplasts before being delivered into the host plant. Protoplast transformation can also be used directly to produce transformed cells and eventually plants as described in panel D. The general procedure of transformation also usually followed by a selection process to select resistant cells and seedlings, and finally a sequencing process to confirm presence of transformed mutants. (**B**) Plant genome editing via *Agrobacterium*-mediated delivery of CRISPR DNA. *Agrobacterium* containing the vectors are transfected into plant cells in the form of calli, embryos, or leaf explants, followed by the selection process to produce genome-edited plants (**C**) Conventional and transient expression approaches for particle bombardment-mediated genome editing via CRISPR DNA, RNA, or RNP delivery. Transformation vectors-coated gold particles are bombarded into plant cells followed by the selection process (**D**) Protoplast transformation with CRISPR DNA, RNA, or RNP. Transformation vectors, protoplasts, PEG, and Ca^2+^ ions are mixed before further selection processes to isolate transformed calli, seedlings, and finally genome-edited plants. (**E**) Two ways to obtain transgene-free mutants. Using the conventional method, a selection agent is used to select resistant calli and transgenic plants. Transformation vectors can be segregated out from the mutant genomes via selfing or crossing. Using the transient method, no selection agent is needed to segregate out the transformation vectors to produce transgene-free mutants. [RNP, ribonucleotide protein].

Given the simplicity, versatility, and efficacy of the CRISPR/Cas9 system, this technology shows great potential for target mutagenesis in various plant species. Despite these advantages and significant developments in the CRISPR/Cas system, continuous efforts to improve its efficiency and practicality in agriculture are still desirable. Figure 2 summarizes the general procedures involved in plant transformation and CRISPR-based plant genome editing.

## 4. CRISPR/Cas9 for Genome Editing in Crops

The CRISPR/Cas9 system has been used in various crops to develop desirable and heritable traits, such as yield improvement, and biotic and abiotic stress management. Table 1 summarizes the applications of the CRISPR/Cas9 for crop improvement.

### 4.1. Abiotic Stress

Climate change leads to various abiotic stresses, threatening agricultural food production worldwide [101]. About 90% of all arable lands are prone to single or multiple abiotic stresses, such as water stress, extreme temperature, and salinity [102]. To survive, plants have evolved various mechanisms to respond to and cope with these stresses [103]. However, the plant stress-responsive and adaptation mechanisms are complex and governed by various genes, posing challenges to developing novel cultivars using the conventional methods [104]. As such, targeted genome editing on a single or multiple target sites through the CRISPR/Cas9 system could be a promising approach to developing abiotic stress-resilient crop varieties [25].

The CRISPR/Cas9 approach has been exploited to improve crop survival under adverse environmental stresses. For example, Zhang et al. [105] developed salinity-resistant rice through the CRISPR/Cas9 approach. By knocking out the *OsRR22* gene, the authors found that the generated rice showed better plant growth than wild-type under salinity conditions [105]. A recent study indicated that *OsNAC041* is a critical transcription factor involved in the salt stress response in rice. A targeted *osnac041* mutant obtained using the CRISPR/Cas9 method showed a higher plant height than wild-type [106]. Other studies demonstrated that members of the AP2/ERF domain containing the RAV (related to ABI3/VP1) transcription factor family are involved in salinity stress adaption [107,108]. For instance, when the rice was exposed to salt stress, the *OsRAV2* gene was activated. To determine the role of the GT-1 element in the *OsRAV2* gene, Duan et al. [109] designed a sgRNA targeting the GT-1 region of the promoter. They found that the mutant lines could not express the OsRAV2 gene under salinity conditions, confirming the importance of this gene in response to salinity stress. A similar finding has been reported by Liu et al. [110], where the CRISPR/Cas9-mediated *OsGTγ-2* knockout lines showed salt-hypersensitive phenotypes. Besides rice, the CRISPR/Cas9 genome editing technology has also been applied to other crops, such as wheat [111], soybean [112], maize [113], and tomato [114].

Drought stress disturbs physiological and biochemical processes in plants, limiting plant growth and yield [115]. Several genes and phytohormone signaling pathways have been shown to play critical roles in drought stress responses. Of these, abscisic acid (ABA) is a central regulator of water use and coordinates the plant’s responses to drought stress. Hence, several studies have been conducted to improve drought tolerance in crops by targeting the genes involved in ABA signaling. For example, Zhang et al. [116] determined the role of *OsABA8ox2*, which encodes ABA 8′-hydroxylase, in rice drought tolerance. The authors found that the CRISPR/Cas9-mediated *OsABA8ox2* knockout lines showed increased drought-induced ABA in roots and induced root formation beneficial to drought tolerance. In contrast, overexpressing *OsABA8ox2* in rice suppressed root elongation and exhibited hypersensitivity to drought stress [116]. The ENHANCED RESPONSE TO ABA1 (ERA1), which encodes the β-subunit of the protein farnesyltransferase, was mutated in Japonica rice cv. Nipponbare using the CRISPR/Cas9 system [117]. The rice *osera1* mutant lines showed increased sensitivity to ABA and drought tolerance through stomatal regulation, suggesting that ERA1 could be a potential candidate gene for enhancing drought tolerance in crops. Another study by Yin et al. [118] showed that the *OsEPFL9* (Epidermal Patterning Factor like-9) mutants had more than an eight-fold reduction in stomatal density (SD) in the CRISPR/Cas9-edited rice plants. The reduced SD allows the edited rice lines to resist drought stress. Under optimal conditions, a significant reduction in carbon assimilation and conductance and enhanced water use efficiency (WUE) was observed when SD was reduced by 50% in barley and wheat [119,120]. Likewise, in well-watered conditions, a CRISPR-based knockout of grapevine *VvEPFL9-1* reduced SD by 60% and caused reduced carbon assimilation as compared to WT [121]. In tomatoes, *slmapk3* mutants generated through CRISPR/Cas9 showed that *SlMAPK3* is involved in drought response, and the *slmapk3* mutants showed more severe wilting symptoms and suffered cell membrane damage under drought stress [122].

Some studies used the CRISPR/Cas9 technology to reduce mineral toxicity. For example, Nieves-Cordones et al. [123] developed low cesium-containing rice plants by inactivating the K+ transporter OsHAK1 using the CRISPR/Cas9 system. In rice, knocking out OsARM1 and *OsNramp5* showed improved arsenic tolerance [124] and low cadmium accumulation [125], respectively. Another example of increasing plant stress resistance was shown by Shao et al. [126], where the authors developed a semi-dwarf variety of bananas using the CRISPR/Cas9 system to disrupt the genes responsible for the gibberellin biosynthesis. As a result, the developed bananas are more resistant to storms and heavy wind. Besides generating knockouts on the susceptible genes, genome-editing tools can also be used for knock-ins of a desirable gene. For instance, Shimatani et al. [127] used CRISPR/Cas9 to insert a maize promoter before the drought tolerance gene, *ARGOS8*. Consequently, the edited maize crops showed a greater grain yield during water stress.

These studies demonstrated that the CRISPR/Cas system could edit the plant genome, allowing us to investigate the role of genes involved in response to abiotic stresses. However, reports on targeting abiotic stress tolerance genes are scarce, primarily due to the complexity associated with abiotic stress tolerance, often involving the modulation of several genes to alter the trait of interest.

### 4.2. Biotic Stress

Plants are constantly plagued by pathogens, such as viruses, bacteria, and fungi, which can significantly reduce crop quality and yield [128]. The majority of disease-resistant crops against non-viral diseases are produced through genome editing and targeted mutagenesis of genes that negatively influence defense [129]. While few such genes are available for increasing disease resistance, many of these loci have already been successfully exploited for increased resistance.

In rice, genome editing has shown a remarkable result in combating diseases using CRISPR/Cas9. Most pathogens use the sucrose transporters that are encoded by the *SWEET* gene family in many plants [130]. In two experiments, CRISPR/Cas9 was utilized to target the promoter region of a few *OsSWEET* genes to develop resistance against bacterial leaf blight [131,132]. Knockout of the *OsERF922* gene that expresses ethylene response in the plant using CRISPR/Cas9 reduced the effect of leaf blast disease, thereby enhancing its tolerance toward it [133]. Additionally, CRISPR/Cas9 editing of the eukaryotic elongation factor, *eIF4G*, in rice resulted in plants that were immune to the rice tungro virus [134]. The infected CRISPR-edited plants contained no detectable viral proteins and produced better yields than wild-type plants.

The advancement of the CRISPR/Cas9 system has furthered the development of resistance to multiple diseases at the same time. Engineering the broad spectrum of disease resistance in staple crops on a large scale could provide a single solution to several diseases that are affecting crop production [131]. The editing of *bsr-k1*, a rice gene that binds to and increases the turnover of defense-related genes [135], is an example of this strategy. By “turning off” these critical defense genes, edited rice plants were resistant to both leaf blast and bacterial leaf blight. When challenged with rice leaf blast in the field, the transgenic lines show a greater yield of 50% more without affecting other agronomic features [135]. Likewise, the same strategy has also been applied to other crops for disease resistance. For example, broad-spectrum resistance was obtained by altering a single locus in tomatoes [136]. The *SlDMR6-1* mutations by CRISPR/Cas9 in the edited lines maintain an increased salicylic acid level in the plant with a significant reduction of disease symptoms and pathogen abundance, gaining resistance to *Pseudomonas syringae*, *Phytophthora capsici*, and *Xanthomonas* spp. [136]. In barley, CRISPR/Cas9-mediated editing of *MORC1*, a defense-related gene, increased resistance to barley powdery mildew *and Fusarium graminearum* [137]. In addition, the authors showed that the edited barley plants had lower levels of fungal DNA and fewer lesions.

In some species, targeting homologs of Mildew-resistance Locus (MLO) and other loci enhanced the resistance to these fungal infections. By concurrently targeting the three homologs of the MLO, TaMLO-A, TaMLO-B, and TaMLO-D, CRISPR/Cas9 can increase the resistance of wheat to powdery mildew [49]. Another example is the Tomelo transgene-free tomato, which is resistant to powdery mildew disease and was produced by targeting *SlMlo1* gene using CRISPR/Cas9 [138]. Zhang et al. [139] changed the three homologs of the wheat *TaEDR1* gene simultaneously using CRISPR/Cas9 to improve resistance to powdery mildew disease. In grapevine, targeting of the MLO homologs boosted the resistance to powdery mildew, whereas the edited line of grapevine had a two-fold reduction in powdery mildew sporulation [140]. In other efforts, knockout of the 14-3-3 c and 14-3-3 d protein simultaneously, a negative regulator of disease response, in cotton enhanced resistance to *Verticillium dahliae* [141]. The edited cotton showed fewer disease symptoms and lowered pathogen presence compared to control [141].

### 4.3. Yield

One of the essential keys to sustaining food production is crop yield. It is the most direct means to address the ever-rising food demand from a growing population. However, crop yield is a complex trait regulated by many factors. Therefore, much research has been done to identify the quantitative trait loci (QTLs) associated with morpho-agronomic and yield-related traits in various crop plants [142].

One way genome editing can increase crop yield is to eliminate genes that have a detrimental impact on yields, such as genes limiting grain size and weight [143,144]. In one recent example, CRISPR/Cas9 was used to individually knock out the genes of four negative yield regulators (*Gn1a*, *DEP1*, *GS3*, and *IPA1*) in the rice cultivar Zhonghua 11. Each of the individual knockout mutants, *Gn1a*, *DEP1*, and *GS3*, showed increased yield characteristics in the T_2_ generation [145]. Similarly, Xu et al. [146] used a CRISPR/Cas9-mediated multiplex genome-editing technology to knock out three main rice negative regulators of grain weight (*GW2*, *GW5*, and *TGW6*) simultaneously, and the resulting mutants had a considerable increase in thousands of grain weights. In another study on wheat, CRISPR/Cas was used to knock out the three homoeoalleles of *GASR7*, and the mutant plant produced a much heavier kernel weight when compared to the wild-type wheat plants [147]. Besides grain, targeting a tomato cis-regulatory region in the CLAVATA-WUSCHEL stem cell circuit (CLV-WUS) using CRISPR/Cas9 resulted in an edited tomato with an increased number of locules (seed compartments) and bigger fruit size [148].

Alternatively, genome editing can also influence crop yield through other strategies. CRISPR/Cas9 technology was employed in maize to create high amylopectin variants from superior cultivars by knocking out the waxy gene [149]. The edited maize cultivars yielded 5.5 bushels per acre more than conventionally bred high amylopectin varieties. Furthermore, they could be developed in a shorter time, demonstrating the feasibility of genome editing in particular specialized applications [149]. Furthermore, reducing the ABA response of rice plants can also enhance the yield. Rice plants with simultaneous mutations of class I PYL genes (encoding receptors for ABA) using CRISPR/Cas9 had better yields than the control [95]. Under well-watered conditions, triple knockout of *PYLs 1,4,6* resulted in a 30% increase in yield [95]. It is interesting to see how these ABA-encoding PYL genes affect yield under less-optimal conditions. A recent study shows that under drought conditions, the wheat *PYL1-1B* (*TaPYL1-1B*) is responsible for increased yield and drought resistance, where it exhibited higher ABA sensitivity, photosynthetic capacity and WUE [150].

A higher yield of tomatoes can also be achieved by modifying the flower repressor gene using CRISPR/Cas9. Knockout of the flowering repressor *SELF-PRUNING 5G* (*SP5G*) gene produced tomato plants that have rapid flowering, which in turn yield earlier with compact determined growth [151]. In contrast, mutations in the *SELF PRUNING* (*SP*) gene changed the plant architecture to a bushier state with more branches [152]. The resultant mutants with two modifications had faster flowering time and earlier fruit ripening than the control lines. In another study, CRISPR-based knockout of tomato *SlAGL6* enhanced yield under heat stress. The tomato agl6 mutants displayed facultative parthenocarpy without any pleiotropic effect and produced seedless fruits of equal weight and shape to WT [153]. Under salinity stress, the CRISPR-edited soybean *gmaitr* mutants yield was much less affected than the WT in plant height, number of pods per plant, and seed weight [112]. The number of studies on plant yield and resilience improvement is expected to grow, in line with the rapid advancement of genome editing tools.

## 5. VOSviewer Bibliometric Analysis

We used ‘Visualization of similarities (VOS) viewer’ version 1.6.17; [154] to visualize and analyze the bibliometric network of CRISPR-related publications extracted from the SCOPUS database for the past 10 years (2012 to 2021). VOSviewer is a handy tool that allows a graphical representation and interpretation of networks representing co-authorship, journals, institutions, or co-occurring keywords based on a selected topic of interest [155].

**Figure 3:**
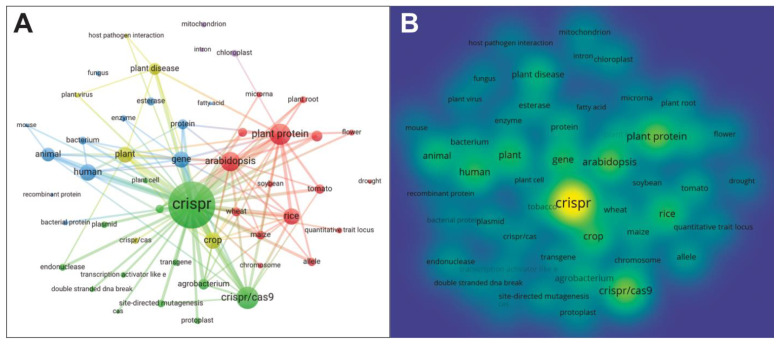
Co-occurrence network of 50 most used keywords in CRISPR-related plant research from 2012 to 2021. (**A**) Network visualization of the keywords based on total link strength. Green, yellow, red, and blue nodes represent four different clusters of keywords identified. A minimum strength of 40 was set for the lines to appear between the nodes. The relatedness of the keywords depends on the number of articles in which they occur together, which is indicated by the size of the nodes/keywords, and the length/thickness of the lines between the nodes. The bigger the nodes/keywords, the larger the weight of the nodes/keywords. The shorter and thicker the lines between the nodes, the more frequently they appear together in the publications. (**B**) Density visualization of the keywords based on occurrences. The density of a keyword depends on the number of keywords around the node. Keywords in the yellow areas indicate a more frequent occurrence in the publications while green areas indicate a less frequent appearance.

Based on our keyword search, more than 5200 scientific papers focused on “CRISPR” OR “genome editing” AND “plants” have been published in the last ten years (2012–2021). We compiled a list of relevant publications with the co-occurrence of keywords in the title, abstract, and keywords sections from all publication types (2012–2021), including journal articles, books, and conference proceedings, to generate bibliographic maps and networks using the software VOSviewer. The criteria were set as follows: the keywords repeated at least five times were selected, singular and plural forms were standardized to singular forms to avoid redundancies, and full names and abbreviations were standardized to full names. Interchangeable keywords (e.g., ’corn’ and maize’) and spelling differences (e.g., ‘colour’ vs ‘color’) were also standardized in the ‘thesaurus’ option before running the bibliographic analysis. Based on these premises, 50 keywords were used and clustered according to their strength of association. Four clusters (sets of closely related nodes) were generated and integrated into a network overlay visualization map. The maps and networks for the analyses are presented in Figure 3. The list of 50 keywords based on their ranking is presented in Table 2.

Table 2 shows that “crispr”, “CRISPR/Cas9”, and “plant protein” are the three most used keywords in CRISPR-related plant research publications. Of several different types of plants/crops (e.g., model plants, food crops, industrial crops, and ornamental plants) [156], only the model plants (Arabidopsis and tobacco) and food crops (rice, tomato, wheat, maize and soybean) are present in the network map. Their total number of occurrences (shown in brackets) are as follows: Arabidopsis (673), tobacco (192), rice (525), tomato (239), wheat (224), maize (213), and soybean (134).

Four CRISPR system-related keywords, “crispr” (2386), “cas” (70), “crispr/cas” (144), and “CRISPR/Cas9” (821), have been identified in the top 50 keywords during the keyword search. These keywords were not grouped in the ‘thesaurus’ option before the analysis since each keyword may represent a unique value. The highest cited keyword, “crispr” (2386 occurrence), may represent the investigation of CRISPR as a biological phenomenon in the bacterial immune system, which later formed the basis of “CRISPR/Cas” technology. After the discovery of the CRISPR/Cas technology, studies on CRISPR as a biological phenomenon have continued to provide knowledge to further improve the CRISPR/Cas system applications.

Further advancements in the CRISPR/Cas systems are oriented to expand its applications to other organisms and cell types and identify other alternatives to Cas9 proteins to improve CRISPR editing scope and specificity [156]. This is reflected by the presence of the keyword “cas” in the network map. The three most distinguishable variants of Cas proteins identified so far are Cas3 in type I systems, Cas9 in type II systems, and Cas10 in type III systems [157]. In addition, many other Cas proteins have also been harnessed to expand the CRISPR/Cas targeting scope, including Cas9, Cas12a, and Cas13 variants and orthologs. The expanding list of these Cas proteins and their applications has been covered extensively in recent reviews [64,158,159,160,161].

The closeness of the keyword “CRISPR/Cas9” with its surrounding keywords, such as “chromosome”, “gene”, “transgene”, “site-directed mutagenesis”, and “crop” indicated the application of the CRISPR/Cas system for the past decade as genome editing tools in crops, allowing specific and targeted changes in the gene of interest. A key approach in plant engineering for the past few decades has involved the integration of specifically assembled DNA cassettes or foreign genes into the host plant. The ability to express non-native segments of DNA in certain plants or crops resulted in novel plants with desirable traits such as herbicide resistance, pest resistance, and disease resistance [162]. It is also worth noting that transgene-free plants produced by genome editing using the CRISPR/Cas-based system (e.g., site-directed-nuclease-1 (SDN-1) type) is rapidly becoming its main selling point to avoid unnecessary regulatory issues and to gain better public perception [163,164]. These two factors are important for genome editing technology to be fully utilized and positively impact on the agricultural sector [165]. This may explain the relatedness of the keywords “crispr/cas” and “transgene” in the network map.

Genome editing reagent delivery into the host genome is a crucial topic in plant genome editing. The two most common ways of transferring a gene of interest into a host plant involved *Agrobacterium*-mediated transformation (AMT) or direct DNA transfer [166]. Both techniques aim to express the integrated transgene, silencing endogenous gene expression or modifying endogenous gene activity or function [167]. Compared to direct DNA transfer, AMT is more cost-effective and accessible to most plant researchers due to its low input (requiring low copies of DNA fragments carrying the gene of interest) and high throughput (high transformation efficiency) [168]. AMT also enables the transfer of large DNA fragments with minimal rearrangement, unlike the direct DNA transfer technique. These qualities made AMT the favored approach for plant transformation. This scenario is reflected by the network map (Figure 3), where the keyword “agrobacterium” stays in proximity to keywords, such as “crispr/cas”, “transgene”, and “crops”.

In contrast, keywords that may be related to physically or chemically direct DNA transfer methods, such as “biolistic delivery”, “gene gun”, “plant bombardment”, “electroporation”, “microinjection”, or “Polyethylene glycol (PEG)-mediated transfer” are not present in the network map. Another topic commonly associated with genome editing and CRISPR tools is using protoplasts (plant cells without cell walls) as a rapid validation system. It provides a platform to test the mutagenesis efficiency of RNA-guided endonucleases, promoters, sgRNA designs, or Cas proteins before the full-scale transformation in the host plant [169]. The popularity of this approach is reflected in the network map in which the keyword “protoplast” is present near “site-directed mutagenesis”, “crispr/cas”, and “transgene” nodes.

Apart from identifying the research “hot spots”, the network map in Figure 3 also revealed gaps in the current state of CRISPR research. For example, the lack of connecting lines and the relatively large distance between “CRISPR” and both plant organelles, “chloroplast” and “mitochondria” indicate the lack of CRISPR application in those organelles, as compared to its already wide application in the nuclear genome in various species. This scenario is probably due to the impermeability of those plant organelles to most RNA and DNA [170]. In addition, the delivery system of CRISPR reagents into the host plant genome remains a challenge in plant transformation. For example, the use of plant bombardment to deliver CRISPR/Cas components may not require a binary vector. However, this technique has other disadvantages, such as random integration into the plant genome, less editing efficiency, and being costlier compared to AMT [171].

Given that one of the main aims of plant genome editing is to mitigate the effects of climate change, it is quite surprising to see the absence of keywords related to environmental stresses (e.g., drought, extreme weather, and elevated temperature or carbon dioxide level). Perhaps these keywords are more used in the other sections (e.g., Introduction or Conclusion sections) and less frequently elaborated in detail in the sections extracted for this analysis (i.e., Title, Abstract, and Keyword sections). In summary, it is possibly safe to assume that the first decade of CRISPR/Cas research may have focused on the ‘foundation’ of the CRISPR/Cas editing system by making various technical improvements in its applications (e.g., the discovery of different Cas proteins, improvements on the delivery system, and evaluation of altered DNA and possible off-target mutations).

## 6. Limitations and Challenges

Genome editing technologies have been employed to make precise changes in plant genomes. They significantly impact both fundamental research and agricultural improvement [172]. Recent modification approaches, particularly CRISPR/Cas, have increased the effectiveness and feasibility of genome editing without the need for incorporating foreign DNA. However, there are still significant obstacles to these technologies in terms of efficient and practical application in crop improvement. One prominent limitation is the off-target effect of these technologies that rely on using SSNs for targeted disruption, insertion, or replacement of selected loci [173]. While most off-targets are caused by identical sequences homologous to the targeted sequence, these effects can also occur in the region close to the target site with unrelated sequences [174]. Efforts have been made to reduce the off-target effects, especially in the CRISPR/Cas system. For instance, many different alternatives of the traditional Cas9 protein have been introduced and developed for higher efficiency and lower off-target effects [175,176,177,178,179,180]. Others, such as base editors that allow for exact nucleotide modifications, epigenome modifiers that change DNA confirmation and related expression levels, and prime editing for precision insertion of small DNA segments, are all prospective options [181,182,183].

Another major challenge of utilizing genome editing technologies to develop improved crops is the stringent regulatory frameworks and extensive risk assessment procedures on GM crops [184]. Most nations have biosafety frameworks in place to govern GM crops generated using recombinant DNA technology. These biosafety frameworks often draw on the fundamental concepts for food safety and the environmental risk assessment of conventional GM crops inserted with foreign gene(s) with desired characteristic(s) [185]. However, with the advent of the gene-edited crop, it is necessary to reassess the present definition of GM crops and the accompanying regulatory frameworks, because different gene editing techniques may introduce different types of alterations in the plant genome. For example, SDN-3 mutation is more similar to the conventional recombinant technique, which introduces a whole transgene into the plant genome, therefore the final product is usually considered a GMO in many nations. In contrast, the SDN-1 can introduce single base substitutions, and in certain cases without the need for introducing DSB. As a result, the genetic features of certain gene-edited crops may differ from conventional GM crops, therefore requiring a case-by-case approach to assess the risk associated with each individual product of the genome editing event [186].

Currently, there is no common regulatory approach at the international level for genome editing because of the continuous debate over the similarities, and differences between gene-edited crops and conventional GM crops. Hence, many countries do not have a clear regulatory policy on the gene-edited crops produced, which further impedes the development and implementation of these improved crops in the field. Nevertheless, the broad use of gene-editing technology poses major technological problems for regulatory bodies to identify and distinguish the regulated crop, as it can be difficult to distinguish the naturally occurring edited events in the plant genome from artificial means. Therefore, an agenda supported by various entities such as experts, associations, regulators, and researchers are needed to address these complex issues and concerns raised by gene-editing in the plant for the benefit of all [187].

## 7. Conclusions and Future Prospects

Genome editing technologies can potentially improve plant agriculture and food production to feed the world’s growing population. Due to their efficiency, ease of engineering, and robustness, CRISPR/Cas systems have revolutionized plant genome engineering and globalized its applications. The current consensus is that CRISPR/Cas systems have the potential to improve plants and crops in various ways, such as increasing crop quality and yield, introducing abiotic stress-resistant traits (such as drought-, herbicide-, and insecticide-resistance), improving food safety by removing the need for an antibiotic-resistant marker, and prolonging food product shelf-life.

The main findings from the bibliographic analysis can be summarized as follows: (1) CRISPR/Cas systems are mainly used for nuclear genome editing. In addition to the nuclear genome, further development and applications of the CRISPR/Cas tools in the plant organelles (i.e., mitochondria and chloroplast genomes) are expected to increase as the technology advances, (2) most CRISPR/Cas editing so far is done on model plants (e.g., Arabidopsis and tobacco) or food crops (e.g., rice, tomato, and wheat). Discovery of novel Cas variants and orthologs and other CRISPR reagents should further expand the targeting scope of CRISPR/Cas systems to other types of plants/crops irrespective of species, (3) the issue of ‘transgene’ usage is one of the most widely discussed in the field of genome editing. Emerging studies on novel genome editing tools are focused on transgene-free editing, which are deemed to be more ‘regulatory-friendly’ and may attract improved public approval, and (4) the research publications are mostly focused on technical advancement in CRISPR systems (e.g., types of editing, targeting scope expansion, types of genomes targeted, and its delivery system) as portrayed by the frequency and relatedness of the extracted keywords in the network map (Figure 3).

Keywords related to regulatory, biosecurity, policymaking, and public acceptance issues are not present in the 50 most used keywords. Keywords related to climate change were also absent from the extracted sections. Despite this, climate change is one of the main driving forces for agricultural innovations to improve food sustainability and security. Regulatory approval and public opinion are also among the key deciding factors for genome-edited plants or crop adaption and commercialization [165,188]. The expanding gap between the fast-paced advances of CRISPR/Cas systems and the surrounding issues related to its regulation, adoption, and public acceptance should not be neglected if the potential of the technology in agriculture and food production is to be fully realized.

The present status of CRISPR technology allows for a wide range of applications aimed at increasing plant yield, disease resistance, and resilience to environmental changes. However, various technological advancements are still required, including precise editing and direct delivery of gene engineering reagents. One way to improve CRISPR delivery into the host genome is to reduce the cargo capacity so that a smaller delivery vehicle can be used to transfer CRISPR proteins through a cell. Another possibility is to use a hypercompact CRISPR CasΦ system. The CasΦ protein (~70 kD) has a molecular weight of half that of Cas9 and Cas12a enzymes [189]. Similar to Cas12a, CasΦ does not require a tracrRNA and produces staggered 5′-overhangs. It also has a minimal PAM requirement, allowing a wider range of target sites in the genome. Despite its low editing efficiency (0.85%), the possibility of using a hypercompact Cas delivery system may pave the way for the use of efficient small gene editors, further expanding the CRISPR editing toolbox.

The bibliographic analysis indicates that the trend of CRISPR/Cas research for the past decade has focused on various ways to improve the CRISPR/Cas functionality (e.g., targeting scope and delivery system). However, only recently, ‘natural brakes’ that could switch off the CRISPR/Cas activity when needed have been discovered. These tools are known as ‘anti-CRISPR’ technology, which uses phages and other mobile genetic elements that express anti-CRISPR proteins (Acrs). These proteins may nullify CRISPR/Cas activity by blocking Cas from binding or cleaving nucleic acid substrates [190]. Future improvements on these ‘natural brakes’ allow for more customized control of plant genome editing and expression, a needed innovation to improve the robustness of the existing CRISPR/Cas toolbox.

The recent development of biotechnologies and the production of novel crop varieties may benefit agricultural efficiency in the face of climate change. Establishing a technology adoption system across multiple farmlands is important to fully realize the potential benefits of these technologies and crop varieties. One of the issues towards adopting technology is the insufficient baseline empirical data to model the risks and benefits of sustainable farming across multiple farm types, farm sizes, and environments [191]. As technological developments are rapidly evolving, there is a constant need to deliver broad knowledge of sustainable farming to the public or industry to reduce the uncertainty about biotechnology and facilitate the adoption of agricultural biotechnology. These combined efforts will hopefully bring a paradigm shift in the farmer’s perspective on sustainable farming and work towards the common goal of food security.

## Figures and Tables

**Table 1:** Examples of CRISPR/Cas9 applications for crop improvement.

Improvement	Trait	Crop	sgRNA Target Area	Type of Editing	Target Area	Result	References
Abiotic stress resistance	Drought	Chickpea	cDNA	Frameshift deletion	*Coumarate ligase (4CL)* and *Reveille 7 (RVE7)*	Enhanced tolerance	[85]
	Cold	Rice	cDNA	InDel mutation	*OsMYB30*	Improved tolerance	[86]
	Herbicide	Maize	cDNA	Base editing	*ZmALS1, ZmALS2*	Plants with Sulfonylurea herbicide-resistant	[87]
	Salinity	Tomato	DBD domain of cDNA	49-bp deletion	*SlARF4*	Enhanced salinity tolerance	[88]
	Heavy metals	Rice	cDNA	Downregulation	*OsNramp5*	Decreased cadmium accumulation	[89]
	Heat	Tomato	cDNA	1-bp insertion 4-bp deletion	*SlMAPK3*	Enhanced heat tolerance	[90]
Biotic stress resistance	Viral disease	Barley	Coding sequence	Base editing	MP, CP, Rep/Rep, IR/Virus genome	Resistant plants	[91]
	Fungal disease	Rice	Genome	80-bp insert	ALB1, RSY1/ Fungal gene	Improved resistance to rice blast	[92]
	Bacterial disease	Tomato	JAS domain C-terminal	Deletion	*SIDMR6-1*/Host S-gene	Resistant plants	[93]
	Insect pest	Soybean	Coding region	1-bp and 33-bp deletion	*GmUGT*	Enhanced resistance to *Helicoverpa armigera* and *Spodoptera litura*	[94]
Plant/crop quality	Crop growth	Rice	cDNA	Frameshift	*PYL1–PYL6* and *PYL12(gp-1)*, *PYL7–PYL11* and *PYL13(gp-2)*	Improved plant growth and grain productivity	[95]
	Crop yield	Wheat	cDNA	10-bp deletion	*TaCKX2-1*, *TaGLW7*, *TaGW2*, and *TaGW8*	Improved grain yield	[96]
	Crop nutrition	Rice	Genomic Safe Harbor	5.2kb insertion	5.2 kb carotenoid cassette insertion	Increased β-carotene content	[97]
	Grain size	Rice	cDNA	InDel mutation	*OsGS3*	Increased grain size	[98]
	Grain number	Rice	cDNA	InDel mutation	*OsGn1a*	Increased grain number	[98,99]
	Fruit size	Tomato	Promoter	85-bp deletion	*SlENO*	Enhanced fruit size	[100]

**Table 2:** A list of 50 most frequently occurring keywords in CRISPR-related plant research publications from 2012 to 2021. The ranking is based on the number of occurrences in the publications. Total link strength indicates the number of publications in which two keywords occur together.

Rank	Keyword	Occurrences	Total Link Strength	Rank	Keyword	Occurrences	Total Link Strength
1	crispr	2386	6260	26	chloroplast	146	384
2	CRISPR/Cas9	821	1873	27	plasmid	146	499
3	plant protein	769	2385	28	crispr/cas	144	267
4	arabidopsis	673	2111	29	transgene	141	542
5	human	535	1629	30	protoplast	140	494
6	crop	531	1450	31	soybean	134	453
7	rice	525	1462	32	enzyme	128	437
8	gene	514	1677	33	flower	124	427
9	plant	512	1597	34	quantitative trait locus	123	405
10	animal	409	1287	35	transcription activator like effector nuclease	117	494
11	plant disease	335	991	36	chromosome	110	376
12	transcription factor	292	1008	37	microrna	110	387
13	agrobacterium	282	1026	38	mitochondrion	108	285
14	protein	262	938	39	double stranded dna break	105	440
15	tomato	239	772	40	plant cell	105	375
16	wheat	224	690	41	bacterial protein	99	414
17	plant leaf	220	814	42	plant virus	96	310
18	maize	213	742	43	fungus	91	314
19	allele	195	708	44	drought	76	211
20	esterase	193	398	45	intron	75	156
21	tobacco	192	699	46	host pathogen interaction	72	267
22	bacterium	181	625	47	cas	70	292
23	site-directed mutagenesis	170	630	48	mouse	60	215
24	endonuclease	155	687	49	fatty acid	54	159
25	plant root	152	498	50	recombinant protein	53	192

## Data Availability

Not applicable.
